# Gelatin sponge as a rare and forgotten cause of early-onset neurological deficit post osteotomy of thoracolumbar kyphosis—A case report and review of literature

**DOI:** 10.1016/j.ijscr.2020.09.113

**Published:** 2020-09-24

**Authors:** Didik Librianto, Fachrisal, Ifran Saleh

**Affiliations:** a Consultant Orthopaedic Spine Surgeon, Department of Orthopaedic & Traumatology, Fatmawati Hospital, Jalan RS Fatmawati No. 4, Cilandak, Jakarta Selatan, Jakarta, 12430, Indonesia; b Consultant Orthopaedic Spine Surgeon, Department of Orthopaedic & Traumatology, Cipto Mangunkusumo National Central Hospital and Faculty of Medicine, Universitas Indonesia, Jalan Diponegoro No. 71, Jakarta Pusat, Jakarta, 10430, Indonesia

**Keywords:** Postoperative neurological deficit, Absorbable gelatin sponge, Spinal cord compression, Case report

## Abstract

**Introduction:**

Neurological deficits complicating the correction of spinal deformity usually occur acutely during surgery or immediately after surgery. Absorbable gelatin sponges have been used to control bleeding and prevent undesired event during and after spinal surgery since more than 50 years ago. However, its potency of osmotic expansion within an enclosed space containing neural tissue can cause compression of the spinal cord. We presented a rare, early-onset postoperative neurological deficit caused by the use of absorbable gelatin sponge.

**Method:**

A 27 years old female patient with kyphosis of thoracolumbal vertebrae at the level of Th 10 – L2 presented with neurologic deficit gradually started 24 h after the posterior vertebrae column resection (PVCR), laminectomies, and Smith Peterson (SPO) osteotomy procedure of severe fixed kyphosis correction of deformity in thoracolumbar area.

**Result:**

Immediate surgical exploration within 24 h was performed with the duration of surgery of 1 h. Hematoma and retained sponge were found within the site of laminectomy, compressing the spinal cord. After removal of gelatin sponge and its associated hematoma, the neurological function returned to normal at 48 h postoperatively.

**Conclusion:**

The use of absorbable gelatin sponge for controlling bleeding and preventing adhesions in spinal surgery has the potency of spinal cord compression due to expansion within the enclosed space, therefore a large piece of absorbable gelatin sponge should be removed once hemostatic control is achieved and small piece, soaked sponge should be used if the sponge is to be left in place, in order to avoid this complication.

## Highlights

• Postoperative neurological deficits can be early- or late-onset.
• Absorbable gelatin sponge can be used for controlling bleeding.
• Gelatin sponge has the potency of spinal cord compression due to expansion within the enclosed space.
• To avoid complication, small piece and soaked sponge should be used.
• Early-onset postoperative neurological deficit should be treated urgently.

## 1 Introduction

Absorbable gelatin sponges have been used to control bleeding during spinal surgery since more than 50 years ago. In addition to control bleeding, absorbable gelatin sponge is used to prevent the development of laminectomy membrane, a well-organized fibrous tissue binding together the dura, nerve roots, and erector spinae muscles, a consistent finding at the laminectomy site [1,2]. Absorbable gelatin sponge is an inert material and is supposedly to be resorbed by the fifth week [2].

The complication after spinal surgery is defined as early-onset or delayed-onset (at 72 h or later after surgery) complication [3,4]. Only few reported a complication of spinal cord compression following the use of absorbable gelatin sponge [5,6]. This was reported to be caused by the osmotic expansion of the absorbable gelatin sponge within the enclosed space. The expansion of the sponge with blood and obstruction of any draining vessel resulted in the obstruction of the neural tissues, including spinal cord [5]. We presented a rare case of spinal cord compression occurred at the early onset after laminectomy and gelatin sponge application. This case report had been reported in line with SCARE criteria [7].

## 2 Patient information

**Fig. 1:**
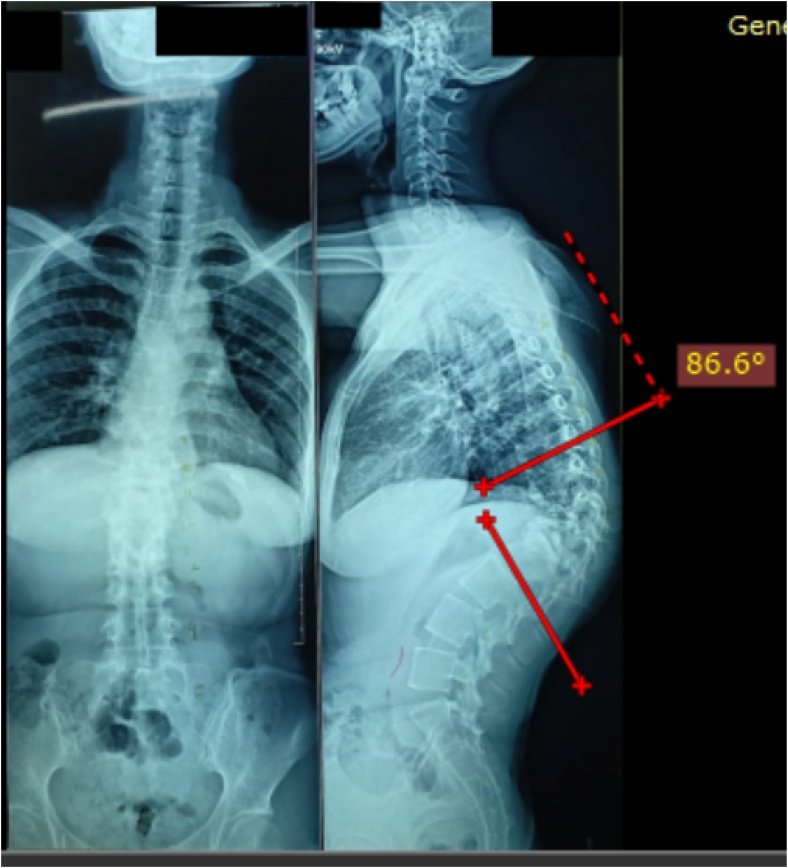
**Patient with Thoracal Kyphosis of 86.6 degree.** The measurement was carried out before the index surgical procedure.

A 27 years old female patient presented with kyphotic deformity of 86.6 degree at the thoracolumbar area from thoracal (Th)10 – Lumbar (L) 2, with destruction of Th11, 12, and L1 due to spine tuberculosis as demonstrated in Fig. 1. Preoperative evaluation showed her neurologic status was without abnormality. She underwent laminectomies, PVCR, one level Smith Peterson Osteotomy (SPO), posterior instrumentation by pedicle screws from Th 8,9,10 and L2,3,4, and fusion by local bone graft.

**Fig. 2:**
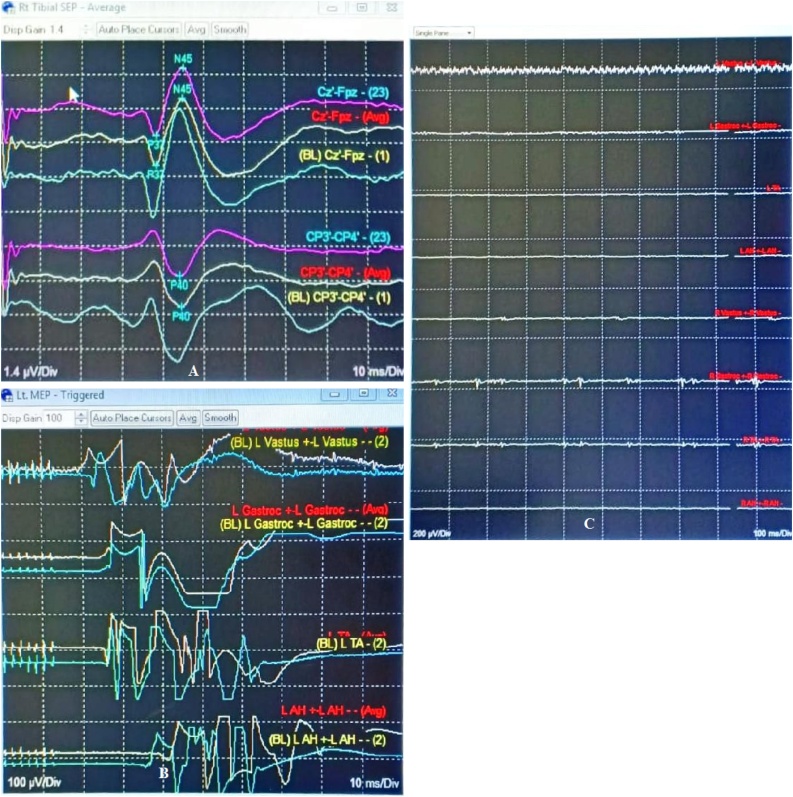
**IONM of the Index Surgery.** (A) SSEP and (B) TcMEP values were as baseline and (C) FrEMG value was without spontaneous activity throughout the operative procedure.

Intraoperatively, the intraoperative nerve monitoring (IONM) showed normal finding without any disturbance, with SSEP (somatosensory-evoked potential) and TcMEP (trans cranial motor-evoked potential) values were as baseline and FrEMG (free-running electromyography) value was without spontaneous activity throughout the operative procedure (Fig. 2). Pedicle screws insertion were confirmed by fluoroscopic guidance and were in good position, the correction was performed by cantilever technique. After the procedure, there were no pathological changes in SSEP, and TcMEP was preserved. A piece of absorbable gelatin sponge approximately in the size of laminectomy defect was applied over the exposed dura in order to control bleeding and prevent adhesion of soft tissues. During surgery the haemodynamic was good. Blood loss during surgery was 1500 cc, and packed cells replacement of 1500 cc was given. The wound was closed and a drain was placed properly beforehand. Five h postoperatively after patient woke up, motoric power grade 5 was scored for both lower extremities with no abnormalities and with good hemodynamic status. Drain output was recorded to be 480 cc for 24 h, with continuous flow and with no obstruction.

Early during postoperative care within 24 h, patient showed gradual neurological deficit. In left lower extremity, the muscle power decreased from grade 5 to 2, whereas the muscle power of right lower extremity was still normal. Within 48 h after surgery, the muscle power score of 2 involved the right lower extremity, following the contralateral side.

## 3 Clinical findings

Within 24 h postoperatively during follow up, the muscle power of left lower extremity was worsened into the score of 2. Subsequently within 48 h postoperatively, the muscle power of 2 involved both left and right lower extremities.

## 4 Timeline

**Table:** 

Time	Clinical Finding	Treatment
Intraoperative	Intact SSEP and TcMEP	PVCR and pedicle screw insertion were performed, absorbable gelatin sponge was applied
Immediately postoperative	Muscle power strength grade 5 for right and left lower extremities	Usual postoperative care
Within 24 h postoperatively	Muscle power strength grade 2 for left lower extremity	Usual postoperative care and close observation
Within 48 h postoperatively	Muscle power strength grade 2 for left and right lower extremities	Usual postoperative care and close observation, CT scan of vertebrae, plan for revision surgery

## 5 Diagnostic assessment

**Fig. 3:**
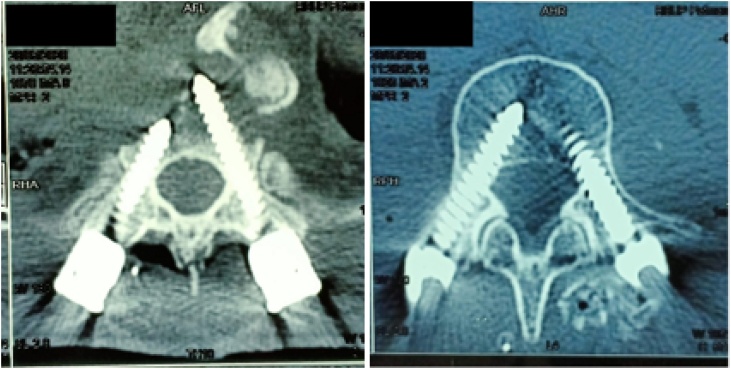
**Postoperative CT Scan of the Vertebrae.** CT scan showed no screw misplacement, thus excluding screw violation as the cause of postoperative neurological deficit.

Postoperative Computed Tomography (CT) was performed to see whether there were screws misplacement causing neurological deficit. MRI was not performed as in our center, it needs more time to perform urgent MRI. As seen in Fig. 3, the CT scan showed no screw misplacement causing any neural tissue impingement from medial breach of the screw. The gradually decreasing motoric strength from normal and normal CT findings lead to the diagnosis of postoperative cord compression caused suspected by hematoma or other material.

## 6 Therapeutic intervention

**Fig. 4:**
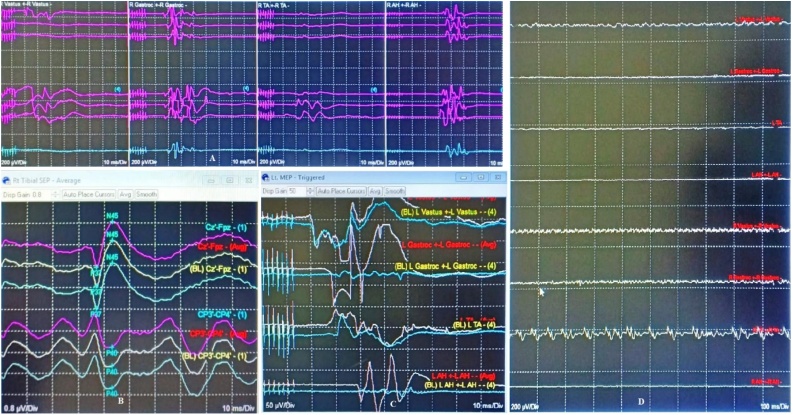
**IONM of the Second Surgery.** Preoperative (second surgery) IONM showed 80% decrease of MEP value from preoperative (first surgery) value. Upon removal of the hematoma and gelatin sponge compression the spinal cord, the SEP and MEP values improved.

Exploration surgery was immediately performed by the previously attending surgeon (D.L) within 24 h to diagnose and manage the postoperative neurological deficit suffered by the patient. IONM showed 80% decrease of TcMEP amplitude value compared to the end of first surgical value (Fig. 4). The duration of surgery was 1 h.

**Fig. 5:**
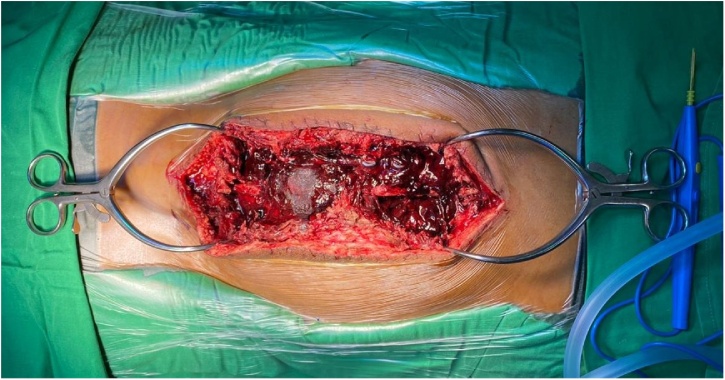
**Exploratory Surgery.** Surgery was performed within 24 h after deterioration of the neurological function. Intraoperatively, engorged, unabsorbable gelatin sponge mixed hematoma was found compressing the spinal cord. Removal of the unabsorbed gelatin sponge and hematoma was carried out.

Intraoperatively on exploration, large hematoma was found in and surrounding the previous laminectomies on osteotomy site. The previously applied absorbable gelatin sponge was found to be liquified and mixed with the blood clot and hematoma, compressing the spinal cord within spinal canal, with no bleeding. Upon removal of the unabsorbed gelatin sponge and the hematoma, the IONM was found to be improved, its value increased almost 80% and the dura was pulsating. The wound was handling by care and drained was placed into properly (Fig. 5).

## 7 Follow up and outcomes

Postoperatively, patient showed improvement in term of neurological function. Patient regained full motor function in both lower extremities. 24 h after surgery, the motor power increased from grade 2 to 4 for both lower extremities. In postoperative day 2, the muscle strength of grade 5 was achieved by both lower extremities. During post-operative care the drainage and patient’s haemodynamic were good. Patient was then allowed to ambulation after 6 days with additional thoracolumbal orthoses with no remained neurological deficit. The postoperative Cobb angle improved into 31 degree.

## 8 Discussion

According to previous research, the incidence of unplanned revision spinal surgery occurring within a week after the index procedure was relatively rare, accounting for 1.12% of cases. The causes of this revision surgery, in order of frequency, were screw malposition, symptomatic epidural hematoma, and inadequate decompression. The screw malposition was the most common cause of revision surgery with the rate of 0.82%, however, the mean time interval of reoperation due to epidural hematoma was shorter, suggesting that this epidural hematoma was more emergent complication than other two common causes of revision spinal surgery [8,9].

The major indication of revision spinal surgery is deterioration of neurological status. Therefore, if patient experiences early-onset motor weakness and/ or decrease in sensory function and/ or loss of anal tone or urinary retention following index spinal surgery, several possibilities must be taken into the consideration, namely screw malposition, intraoperative nerve root injury, vascular compromise, and inadequate decompression. Presence of hematoma is highly suggestive in patient whose symptoms and signs improve after index operation but then experiences early, progressive onset of neurological deficits. In the case of hematoma, early revision surgery for hematoma removal can result in better neurological outcomes. According to the algorithm proposed by Tsai et al. [9], the possibility of screw misplacement in patient developing postoperative neurological deficit can be established if the neurological deficit is the exacerbation of the preoperative symptoms and signs. If after the index procedure, there are persistent neurological deficits which are nearly identical to the preoperative findings, then the possibility of inadequate decompression is highly suggestive as the cause deficits.

During laminectomy, an abundant epidural plexus is usually encountered at the lateral border of the spinal canal. Beside electrocautery, an absorbable gelatin sponge is very helpful in controlling bleeding [10,11]. To prevent and tamponade epidural venous bleeding after wide laminectomy, an absorbable gelatin sponge is usually placed in dorsal spinal canal at the level of laminectomy after the dura is closed [12,13]. It is an effective hemostatic agent. It is widely and commonly used due to its hemostatic ability, antigenicity, and absorbability [6]. Absorbable gelatin sponge is an inert material and is supposedly to be resorbed by the fifth week by phagocytic action of macrophages. It also can undergo rapid absorption by polymorphonuclear leukocytes within days by liquefaction process [2,14].

In our case, the screw misplacement as the cause of postoperative neurological deficits was excluded because immediately after index surgery, there was no exacerbation of the preoperative symptoms and routine postoperative plain radiograph and CT scan showed no violation caused by screw placement. Inadequate decompression as the cause of neurological deficits was excluded because immediately after surgery, the neurologic function of the patient was normal. The probability of hematoma as the cause of progressive neurological deficits was high, and therefore immediate revision surgery was performed in order to achieve better neurological outcomes. In addition to the formed hematoma, the engorged, non-resorbed gelatin sponge can become the culprit because it may absorb blood and surrounded with clots as long as 3 days. It was found that sponge, in its dry state, merely floats. Thus, before use, the sponge must be soaked, can be with thrombin. If the sponge is in its partially moistened state, the air will maintain its pores, which may result in excessive expansion when it contacts with liquid, including blood and tissue fluid. A mass formed by the gelatin sponge which compressed the spinal cord was due to its mixture with the blood, plasma exudate, and probably the early granulation tissue [12]. The explanation of why the applied gelatin sponge caused spinal cord compression is as follow: the gelatin sponge initially swelled as it absorbed blood. Subsequently, when it began to liquify, it exerted an osmotic pressure which attracted more tissue fluid and blood into the sponge, thus filling the compressible space of the sponge. The increasingly swelled absorbable gelatin sponge, being located in enclosed space, acted like an expanding subdural hematoma, compressing more spinal cord over time [6]. This expanding sponge within the enclosed space of spinal canal explained the gradually, progressive deterioration of neurological function.

The obstructed drain as a cause of hematoma development within the laminectomy site was excluded by the observation that the postoperative drain flew continuously. The risk of postoperative epidural hematoma after spinal surgery is increased by three factors: preoperative, intraoperative, and postoperative factors. One of the intraoperative factors is intraoperative bleeding of more than 1000 cc, which occurs in our patient. This might explain the presence of hematoma, other than unabsorbed gelatin material, found within the laminectomy site during exploratory surgery [15].

The engorged gelatin sponge, acting like expanding spinal epidural hematoma, must be rapidly treated with surgical removal because there is relationship between surgical timing and neurological outcome. We performed surgical revision within 24 h after the evidence of deterioration of neurological function. Previous research showed that patients taken to revision surgery within 12 h had better neurological outcomes than patients with identical preoperative Frenkel grade whose surgery was delayed beyond 12 h, showing that rapid diagnosis and emergency surgical treatment maximize the neurological recovery [16].

Based on authors’ knowledge, only few reported the complication of the neurological deficit caused by gelatin sponge. Observation by Dubin et al. showed that in patients with symptom of pain without neurological deficit after laminectomy procedure and supporting MRI findings of mass on laminectomy site and cord compression, the partial resorption of edema, blood, and absorbable gelatin sponge occurred with time, thus diminishing the cord compresstion [12]. However, this observation-only treatment was performed in patient with no neurological deficit. Friedman et al. [17] reported a lumbar cauda equina syndrome associated with the use of gel-foam developed late, 13 days after the index surgery. Upon revision surgery, they found a hard epidural fibrosis admixed with the enlarged gel-foam, and the neurological examination improved after removal of the materials. Alander et al. [5] reported a case of acute quadriparesis after cervical decompression and fusion due to the retained gel-foam compressing the spinal cord. A randomized controlled trial performed by Renkens et al. [18] compared spinal surgery using gelatin-based hemostatic sealant (treatment arm) and Gelfoam–thrombin (control arm) in stopping intraoperative bleeding during spinal surgery. They found that both gelatin-based hemostatic sealant and gelfoam-thrombin were as effective as each other. A randomized controlled trial performed by Renkens et al. [18] compared spinal surgery using gelatin-based hemostatic sealant (treatment arm) and Gelfoam–thrombin (control arm) in stopping intraoperative bleeding during spinal surgery. They found that both gelatin-based hemostatic sealant and gelfoam-thrombin were as effective as each other.

Control of intraoperative bleeding during spinal surgery is important. It can reduce the need of both blood production, intraoperative use of blood salvage device, also reduce the postoperative bleeding. Moreover, rapid and effective hemostasis during spinal surgery will allow control of osseous or epidural bleeding which are not amenable to suture or cautery, allowing the surgeon to have improved visualization of the surgical site [18]. The use of gelatin sponge in controlling bleeding in spine surgery is safe, and its appropriate use must be acknowledged. Before its use, the sponge must be squeezed to expel any trapped air bubbles. Small pieces of sponge should be use as a large piece of sponge will delay its bio-resorption. It is to be soaked before its application and removed once bleeding is controlled if the aim is only to control bleeding. Moreover, large pieces of sponge should not be packed between an interbody bone graft and the spinal cord. If a patient presented with worsening of neurological function postoperatively in which the hematoma formation or gelatin sponge was thought to be the culprit, emergent surgical removal should be carried out to maximize neurological recovery.

## 9 Conclusion

The use of absorbable gelatin sponge for controlling bleeding and preventing adhesions in spinal surgery has the potency of spinal cord compression due to expansion within the enclosed space, therefore a large piece of absorbable gelatin sponge should be removed once hemostatic control is achieved to avoid this complication, and small piece, soaked, sponge should be used if the sponge is to be left in place. If a patient presented with worsening of neurological function postoperatively in which the hematoma formation or gelatin sponge was thought to be the culprit, emergent surgical removal should be carried out to maximize neurological recovery.

## Funding

The authors received no financial support for the research, authorship, and/or publication of this article.

## Ethical approval

The ethical approval was not required for this case report.

## Consent

Written informed consent was obtained from the patient for publication of this case report and accompanying images. A copy of the written consent is available for review by the Editor-in-Chief of this journal on request.

## Author contribution

Didik Librianto: study concept, data collection, data interpretation, and writing the paper.

Fachrisal: data collection, data interpretation and writing the paper.

Ifran Saleh: data collection, data interpretation and writing the paper.

## Registration of research studies

NA.

## Guarantor

Didik Librianto.

## Provenance and peer review

Not commissioned, externally peer-reviewed.

## Declaration of Competing Interest

The authors report no declarations of interest.
